# Intraprocedural hologram support with mixed-reality technique in endoscopic ultrasound-guided biliary drainage

**DOI:** 10.1055/a-2335-6642

**Published:** 2024-06-25

**Authors:** Kazumasa Nagai, Maki Sugimoto, Takasyoshi Tsuchiya, Ryosuke Tonozuka, Shuntaro Mukai, Kenjiro Yamamoto, Takao Itoi

**Affiliations:** 1 13112 Department of Gastroenterology and Hepatology, Tokyo Medical University, Tokyo, Japan; 2 Okinaga Research Institute, Teikyo University, Tokyo, Japan

Endoscopic ultrasound-guided biliary drainage (EUS-BD) is being used increasingly frequently in patients with benign biliary diseases 1 2 . However, puncturing and exploring the intrahepatic bile duct, which runs in a complicated tortuous fashion, can be challenging using two-dimensional (2D) images of EUS and fluoroscopy. Successful EUS-BD is necessary to understand the biliary anatomy, identify the appropriate puncture point, and advance the guidewire smoothly. Thus, it would be ideal to have a device that could confirm the bile duct route with a three-dimensional (3D) device during the procedure. Holograms, which are computer-generated graphics models, have recently been used with mixed reality techniques as a surgical navigation tool 3 4 . Herein we report the first case of EUS-BD using a 3D hologram of the bile duct.

A 26-year-old woman with a history of pancreatoduodenectomy for a solid pseudopapillary neoplasm of the pancreas presented with cholangitis due to a biliojejunal anastomotic stricture. We decided to perform an EUS-guided hepaticogastrostomy. 3D images of the biliary tract were created from magnetic resonance cholangiopancreatography ( Fig. 1 ) using SYNAPSE VINCENT (Fuji Film Medical Co., Ltd., Tokyo, Japan). Data were converted into 3D polygon data ( Fig. 2 ) using the Holoeyes XR system (Holoeyes Inc., Tokyo, Japan) installed on a HoloLens head-mounted display (Microsoft Co., Redmond, Washington, USA) ( Fig. 3 ). Although the bile duct was thin and complicated, the operator wearing the head-mounted display was able to identify the appropriate puncture point from the 3D cholangiogram projected in space ( Fig. 4 ) and successfully complete the procedure ( Video 1 ).

**Fig. 1:**
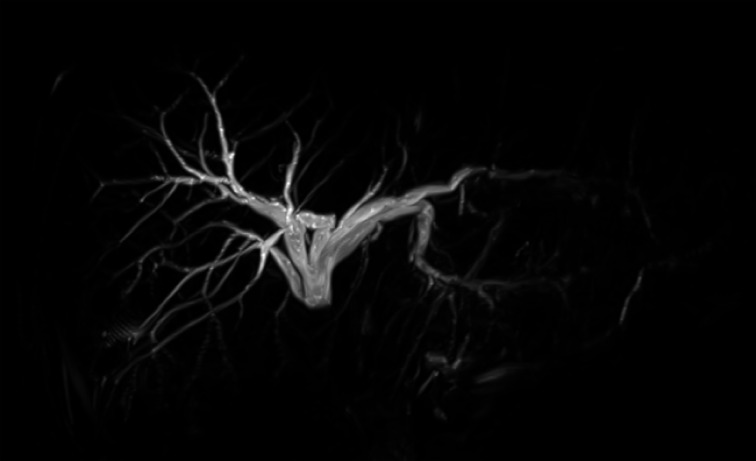
Magnetic resonance cholangiopancreatography (MRCP) image of the biliary tract.

**Fig. 2:**
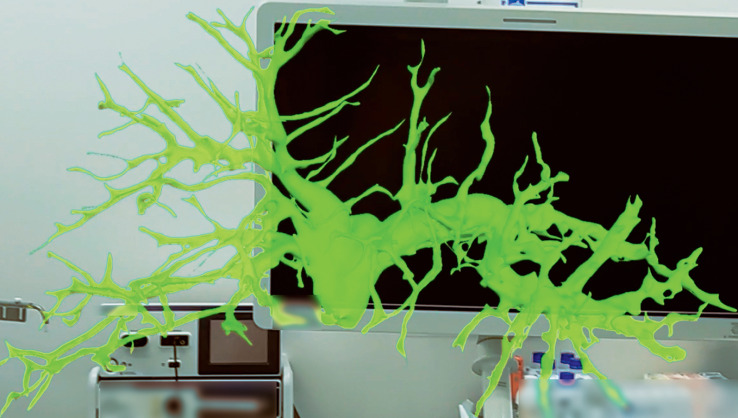
3D cholangiographic image created from MRCP projected on a HoloLens head-mounted display.

**Fig. 3:**
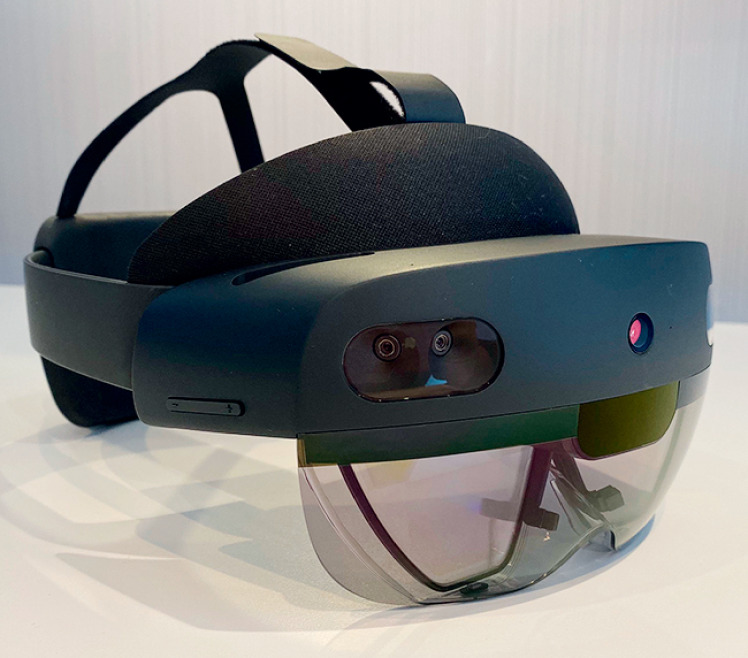
The HoloLens head-mounted display.

**Fig. 4:**
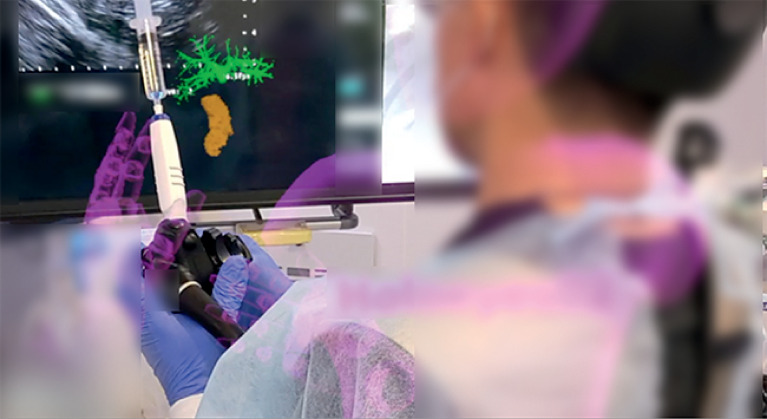
The image shows the operator wearing a head-mounted display, identifying the appropriate puncture point using the 3D cholangiogram projected in space.

Endoscopic ultrasound-guided biliary drainage using a 3D cholangiographic image projected in space as a reference.Video 1

To our knowledge, this is the first report of EUS-BD using a 3D hologram. A 3D cholangiogram may make it easier for some to understand the biliary anatomy than a 2D image. This is an innovative technology that allows EUS-BD to be safely performed.

Endoscopy_UCTN_Code_TTT_1AS_2AD

## Notes

Endoscopy E-Videos https://eref.thieme.de/e-videos
*E-Videos* is an open access online section of the journal *Endoscopy* , reporting on interesting cases and new techniques in gastroenterological endoscopy. All papers include a high-quality video and are published with a Creative Commons CC-BY license. Endoscopy E-Videos qualify for HINARI discounts and waivers and eligibility is automatically checked during the submission process. We grant 100% waivers to articles whose corresponding authors are based in Group A countries and 50% waivers to those who are based in Group B countries as classified by Research4Life (see: https://www.research4life.org/access/eligibility/ ). This section has its own submission website at https://mc.manuscriptcentral.com/e-videos .
